# Supplementary material for: Simultaneous Promotion of Salt Tolerance and Phenolic Acid Biosynthesis in Salvia miltiorrhiza via Overexpression of Arabidopsis MYB12
Source: Int J Mol Sci. 2023 Oct 24;24(21):15506. doi: 10.3390/ijms242115506 (PMC10648190; doi:10.3390/ijms242115506)
Supplement: Supplementary file 1 [file ijms-24-15506-s001.zip › ijms-2617900-supplementary.pdf]

Supporting Information

Simultaneous Promotion of Salt Tolerance and Phenolic Acid Biosynthesis in *Salvia miltiorrhiza* via Overexpression of *Arabidopsis MYB12*

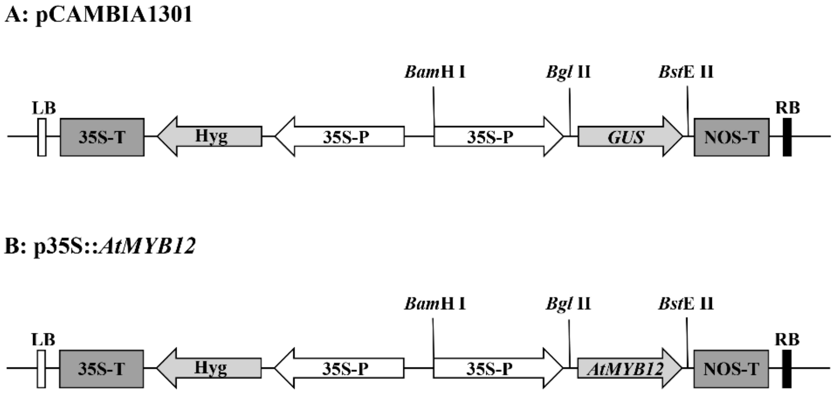

Figure S1. The vector construction map. (A) Construction of the binary vector pCAMBIA1301; (B) *pCAMBIA1301-AtMYB12* binary vector containing Hyg and *AtMYB12* under the control of the 35S promoter. 35S-P, CaMV 35S RNA promoter; 35S-T, CaMV 35S poly A; NOS-T, the 3' terminator region of nopaline synthase; Hyg, Hygromycin B phosphofransferose gene; RB, right border; LB, left border.

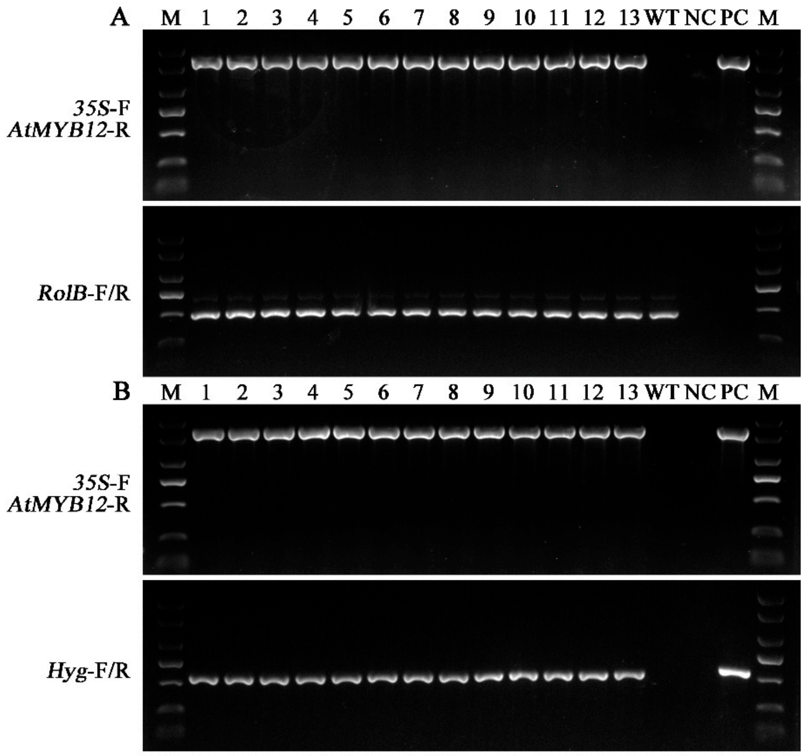

Figure S2. Identification of *AtMYB12* transgenic hairy roots and transgenic plants via PCR. 35S-F, *AtMYB12*-R primers, and *Hyg-F/R* primers were used to identify transgenic positive clones. The *rolB* gene was used to prove that the hairy roots were infected with *Agrobacterium*. WT, wild type; NC, negative control; PC, positive control; M, DL2000 DNA Marker.

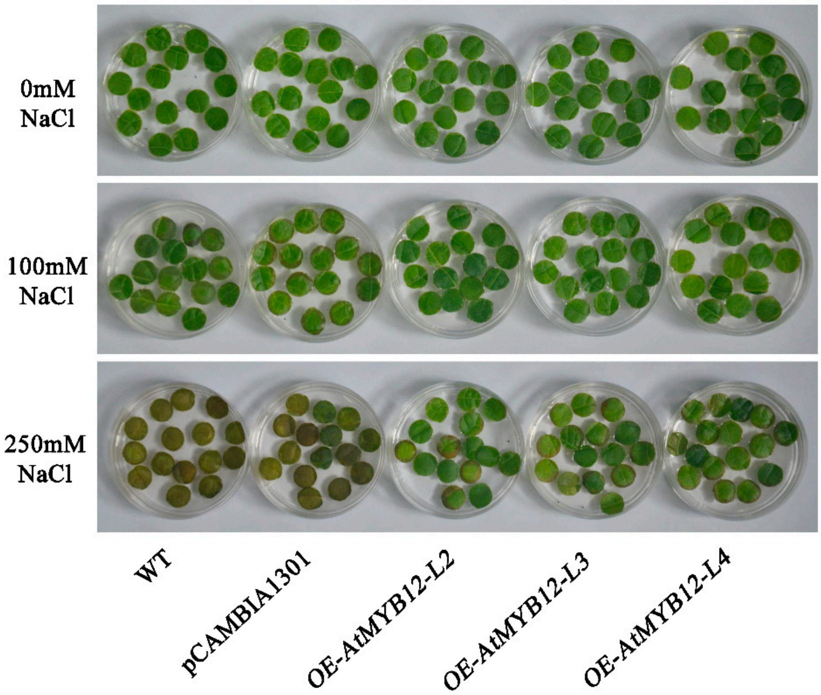

Figure S3. Phenotypic observation of detached leaf discs after treatment with 0 mM, 100 mM, and 250mM NaCl. WT, wild type; pCAMBIA1301, empty vector control pCAMBIA1301 line; OE-*AtMYB12*-L2/L3/L4, pCAMBIA1301-*AtMYB12* transgenic line 2/3/4. Images were taken at 4 DAT (days after treatment).

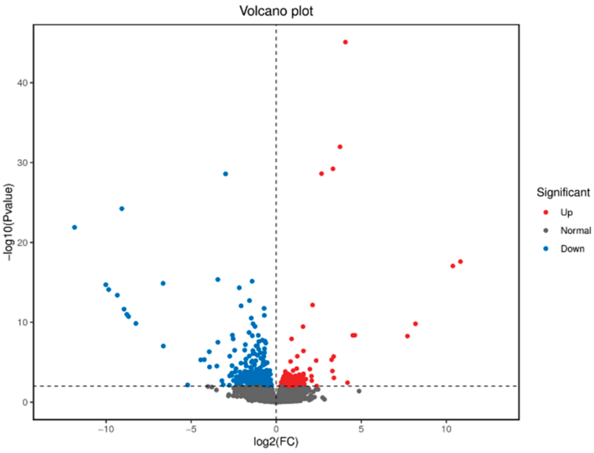

Figure S4. Volcano plot of DEGs of *AtMYB12* transgenic lines versus those of WT plants. Note: in the volcano plot, each dot represents a gene. X-axis: $\log_2$ fold change of expression; Y-axis: $-\log_{10}$ (FDR) or $-\log_{10}$ (P-value). Dots further from to y=0 represent genes with large differences in expression between two samples. Dots further from x=0 represent genes of which the differences are more reliable. Green dots are down-regulated genes, while red dots are up-regulated ones and black dots are genes without significant differences.

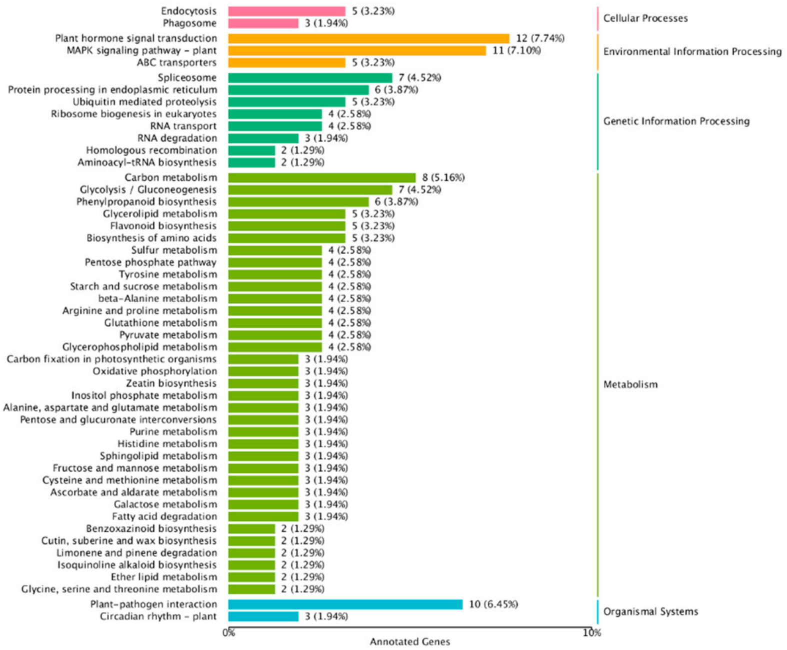

Figure S5. KEGG classification of DEGs of *AtMYB12* transgenic lines versus those of WT plants. Note: Y-axis: KEGG pathway terms; X-axis: number and percentage of genes annotated to the KEGG pathway.

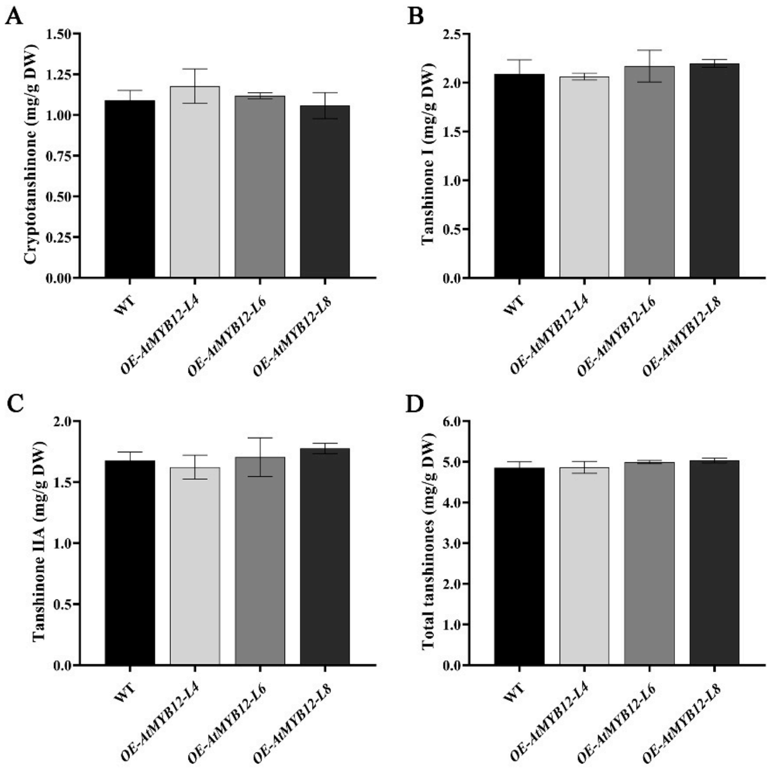

Figure S6. Content of Tanshinone in transgenic hairy roots. WT, wild type; *OE-AtMYB12-L4/L6/L8*,

pCAMBIA1301-AtMYB12 transgenic hairy root line 4/6/8.

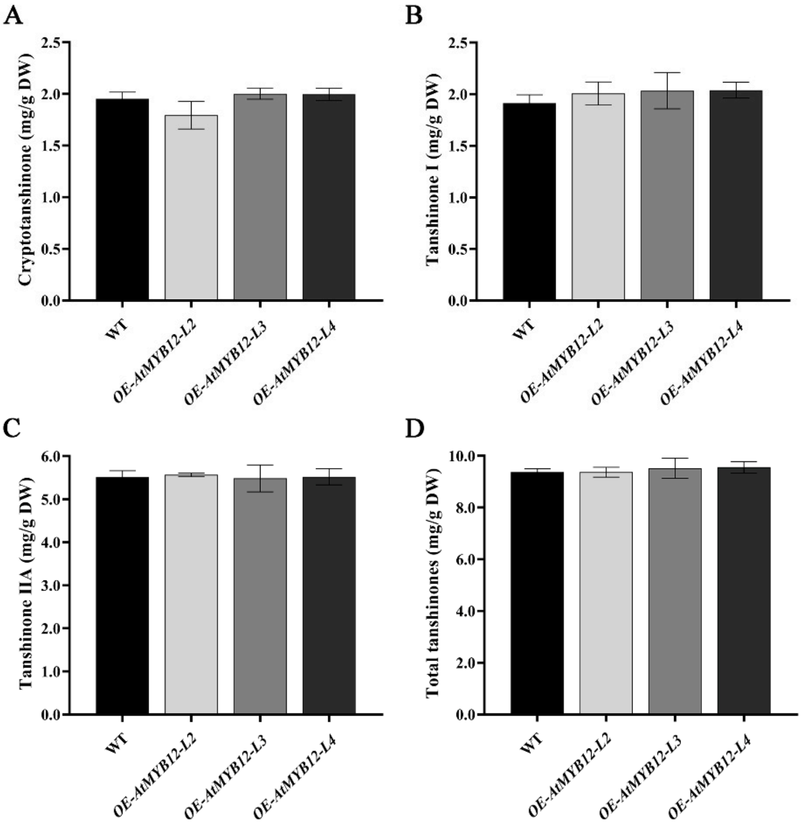

Figure S7. Content of Tanshinone in transgenic *S. miltiorrhiza*. WT, wild type; *OE-AtMYB12-L2/L3/L4*, *pCAMBIA1301-AtMYB12* transgenic plant line 2/3/4.

Table S1. Primers used in this study

Primers	Sequence (5' to 3')
AtMYB12-F	ACTCTTGACCATGGTAGATCTATGGGAAGAGCGCCATGTTGCGA
AtMYB12-R	GGGGAAATTCGAGCTGGTCACCTCATGACAGAAGCCAAGCGA
35S-F	AACAGAACTCGCCGTAAAG
Hyg-F	TACACAGCCATCGGTCCAGACG
Hyg-R	TCGGTCAATACACTACATGGCG
rolB-F	GCTCTTGCAGTGCTAGATTT
rolB-R	GAAGGTGCAAGCTACCTCTC
SmPAL1-QF	ACCCCGTCACCAACCACGTGCAGAG
SmPAL1-QR	CGCCCATTGTGAGAGTTCGTTT
SmC4H1-QF	CCAGGAGTCCAAATAACAGAGC
SmC4H1-QR	GCCACCAAGCGTTCACCAAGAT
Sm4CL1-QF	ATTCGCATTCGCATTTCTCGG
Sm4CL1-QR	GCGGCGTAGTGCTTCACCTTT
SmTAT1-QF	CAACTGCTGGTCTTCCACAAAC
SmTAT1-QR	GCGAGCCAAAACGGACA
SmHPPR1-QF	TGACTCCAGAAACAACCCACATT
SmHPPR1-QR	CCCAGACGACCCTTCACAAG
SmRAS1-QF	CGAGATCGCCTACTCCAAGTTCAAG
SmRAS1-QR	AGATGGCGTTACCGAAGTATCCCTG
SmCYP98A14-QF	GGTCTGTACCGTCGTCCTCTTCTCC
SmCYP98A14-QF	CCTTTTCCCAAATACCAGCCTTGT
SmActin-F	AGCACCGAGCAGCATGAAGATT
SmActin-R	AGCAAAGCAGCGAACGAAGAGT

Table S2. Sequencing data statistics

**Samples**	**Clean reads**	**Clean bases**	**GC Content**	**%≥Q30**
MYB12-1	25,728,465	7,687,414,702	50.09%	94.13%
MYB12-2	24,545,482	7,328,477,624	50.32%	93.74%
MYB12-3	22,065,396	6,572,191,864	49.13%	93.77%
p1301-1	21,309,595	6,365,646,636	49.66%	93.63%
p1301-2	25,825,132	7,717,456,100	49.38%	94.05%
p1301-3	23,376,367	6,973,266,094	50.92%	94.28%

Table S3. Statistics on data mapping

**Sample**	**Total Reads**	**Mapped Reads**	**Uniq Mapped Reads**	**Multiple Map Reads**	**Reads Map to '+'**	**Reads Map to '-'**
MYB12-1	51,456,930	44,373,621 (86.23%)	41,925,617 (81.48%)	2,448,004 (4.76%)	23,512,115 (45.69%)	23,857,199 (46.36%)
MYB12-2	49,090,964	39,894,960 (81.27%)	37,446,652 (76.28%)	2,448,308 (4.99%)	21,234,141 (43.25%)	21,665,776 (44.13%)
MYB12-3	44,130,792	37,415,611 (84.78%)	35,414,752 (80.25%)	2,000,859 (4.53%)	19,920,600 (45.14%)	20,029,738 (45.39%)

p1301-1	42,619,190	36,456,538 (85.54%)	34,507,145 (80.97%)	1,949,393 (4.57%)	19,363,471 (45.43%)	19,514,006 (45.79%)
p1301-2	51,650,264	44,483,735 (86.12%)	42,152,619 (81.61%)	2,331,116 (4.51%)	23,614,010 (45.72%)	23,769,404 (46.02%)
p1301-3	46,752,734	40,777,383 (87.22%)	38,293,216 (81.91%)	2,484,167 (5.31%)	21,708,107 (46.43%)	22,159,331 (47.40%)
